# A Single MicroRNA-Hox Gene Module Controls Equivalent Movements in Biomechanically Distinct Forms of *Drosophila*

**DOI:** 10.1016/j.cub.2019.06.082

**Published:** 2019-08-19

**Authors:** A. Raouf Issa, João Picao-Osorio, Nuno Rito, M. Eugenia Chiappe, Claudio R. Alonso

**Affiliations:** 1 Sussex Neuroscience, School of Life Sciences, University of Sussex, Biology Road, Brighton BN1 9QG, UK; 2 Champalimaud Neuroscience Programme, Champalimaud Center for the Unknown, Brasília Avenue, Doca de Pedrouços, 1400-038 Lisbon, Portugal

**Keywords:** *Drosophila*, microRNA, *Hox* genes, *Ultrabithorax*, *Ubx*, behavior, movement, motor neuron, self-righting, adult/larva

## Abstract

Movement is the main output of the nervous system. It emerges during development to become a highly coordinated physiological process essential to survival and adaptation of the organism to the environment. Similar movements can be observed in morphologically distinct developmental stages of an organism, but it is currently unclear whether or not these movements have a common molecular cellular basis. Here we explore this problem in *Drosophila*, focusing on the roles played by the microRNA (miRNA) locus *miR-iab4/8*, which we previously showed to be essential for the normal corrective response displayed by the fruit fly larva when turned upside down (self-righting). Our study shows that *miR-iab4* is required for normal self-righting across all three *Drosophila* larval stages. Unexpectedly, we also discover that this miRNA is essential for normal self-righting behavior in the adult fly, an organism with different morphology, neural constitution, and biomechanics. Through the combination of gene expression, optical imaging, and quantitative behavioral approaches, we provide evidence that *miR-iab4* exerts its effects on adult self-righting behavior in part through repression of the *Hox* gene *Ultrabithorax* (*Ubx*) in a specific set of adult motor neurons, the NB2-3/lin15 neurons. Our results show that *miRNA* controls the function, rather than the morphology, of these neurons and demonstrate that post-developmental changes in *Hox* gene expression can modulate behavior in the adult. Our work reveals that a common *miRNA-Hox* genetic module can be re-deployed in different neurons to control functionally equivalent movements in biomechanically distinct organisms and describes a novel post-developmental role of the *Hox* genes in adult neural function.

## Highlights

• The fruit fly miRNA gene *miR-iab4* controls the same behavior in the larva and adult
• *miR-iab4* exerts its behavioral roles via repression of the *Hox* gene *Ultrabithorax*
• *miRNA/Hox* inputs affect the physiology and not the anatomy of specific motor neurons
• Conditional expression shows a novel role of the *Hox* genes in adult neural function

## Teaser

Issa, Picao-Osorio et al. explore the molecular cellular basis of behavior. Through the combination of gene expression, optical imaging, and quantitative behavioral analyses in *Drosophila*, they show that a single genetic module—including a microRNA and a *Hox* gene—controls functionally equivalent movements in biomechanically distinct organisms.

## Introduction

Movement first emerges during embryonic development. Although in their initial manifestation motor programs typically appear highly uncoordinated, as development proceeds [1, 2, 3, 4, 5] movement sequences acquire a remarkable level of dexterity, enabling the fully formed animal to feed, escape from predators, or find a suitable partner to mate. As such, adequate movement control is key to the animal’s adaptation to the environment.

Given that the circuit components of behavior are built under the influence of genes [6, 7], the question arises as to what extent the genetic make-up of the organism affects the control of its movements. In principle, following the “Brenner paradigm” [6], genetic mutations could affect the control of movement in two fundamentally distinct ways: they could impair the developmental formation of the networks underlying movement control or interfere with the function of the cellular components involved in the physiological regulation of movement. Nonetheless, these two levels of action of the genetic system need not be mutually exclusive (see below).

A convenient experimental system to study the effects of genes on movement control is the fruit fly *Drosophila melanogaster*. Here, following the behavioral genetics approach pioneered by Seymour Benzer and his colleagues [7, 8], it became possible to isolate several genes with associated roles in movement control, including the zinc-finger transcriptional co-repressor gene *scribbler* [9], the cGMP-dependent protein kinase gene *foraging* [10, 11], the Ig superfamily gene *turtle* [12], the phosphatidic acid transporter gene *slowmo* [13, 14], and other genes, such as *pokey* [15], whose molecular functions have not yet been established. Of note is the case of the *Hox* genes, which encode a family of transcription factors key for the correct development of body structures along the main body axis [16, 17, 18, 19], and whose function has been shown to be required for the correct development of the neuromuscular networks underlying larval crawling [20].

Yet much of the genetic dissection of movement control has so far focused on so-called protein-coding genes. Recent work in our laboratory showed that a single non-coding RNA, the microRNA (miRNA) *miR-iab4*, can affect the complex motor sequence that allows the young fruit fly larva to rectify its orientation if turned upside down (self-righting, SR) [21]. SR is an adaptive innate response that ensures an adequate position of the organism in respect to the substrate and is evolutionarily conserved all the way from insects to mammals, including humans [22, 23, 24, 25].

At the molecular level, miRNAs repress gene expression by blocking protein translation or promoting mRNA degradation of their targets [26]. In this respect, our previous work in the larva showed that *miR-iab4* affects larval movement through the regulation of one of its molecular targets, the *Hox* gene *Ultrabithorax* (*Ubx*) [27, 28], whose level of expression in a set of abdominal metameric motor neurons is critical for normal SR behavior [21]. To explore the generality of the effects of miRNA regulation on larval SR movement, we recently conducted a genetic screen that revealed that at least 40% of all miRNAs expressed in young *Drosophila* larva affect SR, demonstrating an unprecedented and widespread role of miRNA regulation in the control of postural adjustments and locomotor behavior [29].

Despite this progress, it is currently unclear whether functionally equivalent movements performed by morphologically distinct organisms rely on common or different genetic operators. Here we investigate this problem by looking at the effects of the *miR-iab4/Ubx* system on distinct developmental stages of the fruit fly including the larvae and adults: organisms with substantially different somatic and neural constitution, biomechanics, behavioral structure, and lifestyle [30, 31].

Through the combination of gene expression, optical imaging, and behavioral analyses, we show that a single genetic module composed of the miRNA *miR-iab4* and the *Hox* gene *Ubx* contributes to the SR response in both *Drosophila* larvae and adults. Our study also reveals a novel neural role of the *Hox* genes in the fully formed adult, suggesting that these key developmental genes perform previously unknown physiological regulatory functions once development has ceased.

## Results

**Figure 1:**
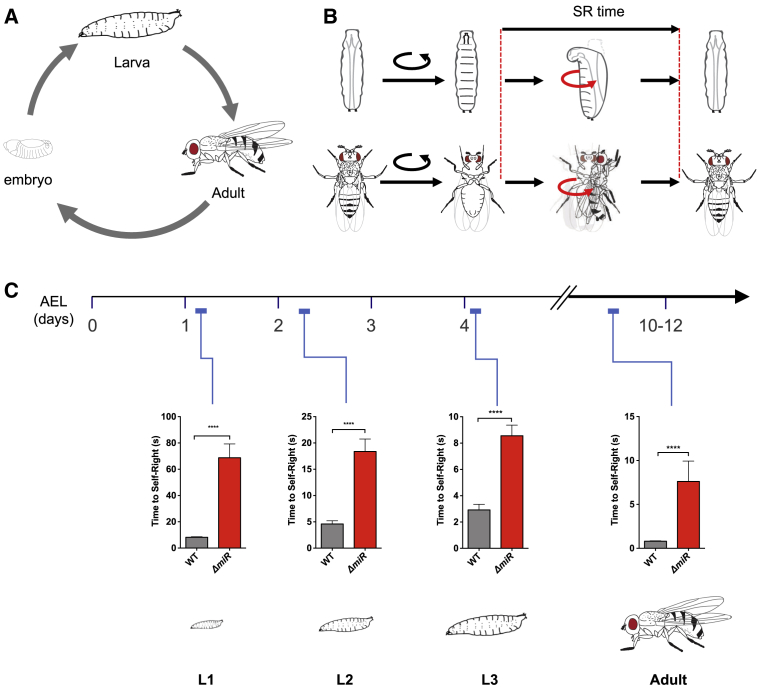
Removal of *miR-iab4/iab8* Disrupts Larval and Adult Self-Righting Behavior (A) *Drosophila melanogaster* life cycle. (B) Diagram of SR behavioral response in larvae (top) and adults (bottom). (C) Quantification of the time required for the successful completion of the SR behavior along larval stages and in the adult (mean ± SEM; N = 63–70 larvae for L1, 27–28 for L2, and 25 for L3, and N = 49–54 adult flies) in wild-type controls (w^1118^, gray) and *miR-iab4/iab8* mutants (*ΔmiR*, red). Analysis of SR behavior throughout development shows that *ΔmiR* mutants have defects across larval stages and in the adult. A nonparametric Mann-Whitney U test was performed to compare treatments; ^∗∗∗^p < 0.001. AEL, after egg laying. (NB: Experiments in adult flies were conducted on wingless specimens, but similar results were obtained using different anesthesia methodologies; STAR Methods.) See also Figures S1 and S3 and Videos S1 and S2.

Our previous work in the young, first instar *Drosophila* larvae showed that ablation of the *miR-iab4/8* locus [32] leads to significant defects in the SR response [21]. To investigate whether *miR-iab4/8*-dependent effects were confined to the L1 stage or had impact throughout larval development, we conducted a series of SR tests in first, second, and third instar larvae (L1, L2, and L3 larvae, respectively) (Figures 1A and 1B). SR was assayed by briefly putting individuals upside down and monitoring the time they took to come back to a normal right-side up position (STAR Methods). miRNA mutant larvae take longer to complete the SR sequence (Figure 1C), indicating that this miRNA system is important for the normal timing of the SR response across all three larval stages.

Like in all holometabolous insects, the *Drosophila* life cycle involves the transformation of the larva into the adult through the process of metamorphosis [33]. Given the substantial anatomical and functional remodeling that metamorphosis imposes on the *Drosophila* soma and nervous systems, genetically induced behavioral defects observed in the larvae may simply disappear in the adult. However, a modification of the SR test performed in the *Drosophila* adult (STAR Methods) reveals that the integrity of the *miR-iab4/8* locus is essential for a normal SR response also in the adult fly (Figures 1C, S1A, and S1B) (Videos S1 and S2). This implies that a common miRNA system controls the same functionally equivalent behavior in *Drosophila* forms that bear different morphological, neural, and biomechanical properties.

**Figure 2:**
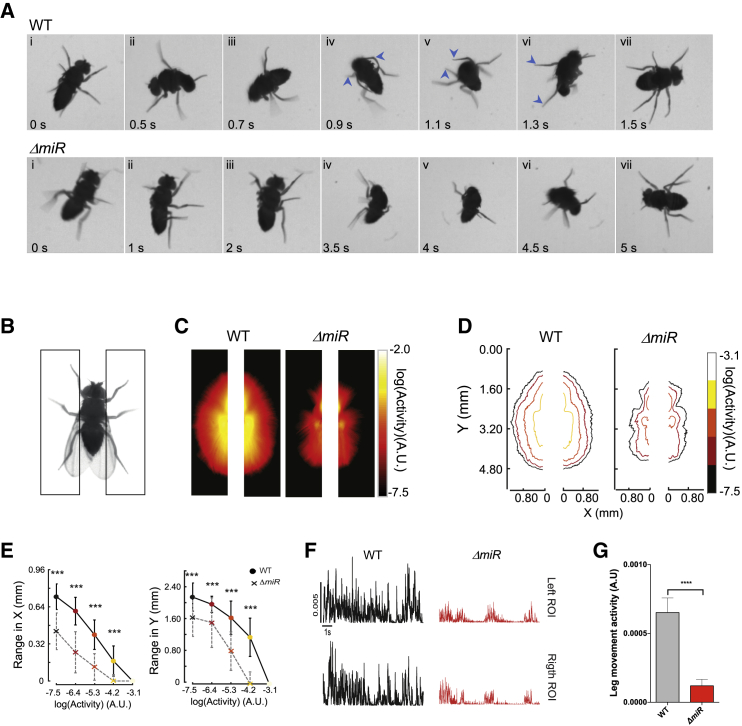
Effects of miRNA Regulation on Leg Movement (A) Description of self-righting (SR) movement in wild-type (top, WT) and miRNA mutant (bottom, *ΔmiR*) adult flies. Manual video analysis shows that SR behavior in adult WT flies involves several components, including (1) detection of abnormal (upside-down) body orientation, (2) horizontal stirring of legs and body, (3) attempts to grab substrate, (4) coordinated movement of left and right third legs anteriorly until substrate is grabbed, (5) lifting of body by the third legs, (6) tilt of the whole body forward, and (7) return to normal position. (NB: Experiments in adult flies were conducted on wingless specimens; see STAR Methods and Figure 1 legend.) See also Videos S1 and S2. (B–G) Quantification of leg movement levels in WT control (w^1118^) and *ΔmiR* flies. Schematic of the paradigm and the regions of interest (black rectangles) drawn to quantify leg movements (STAR Methods) (B). Average heatmaps of leg movements (C) and their corresponding contours (D), and range of movements as defined by the movement contours, in azymuth (right) and elevation (left) axis (E) in *ΔmiR* flies compared to WT (mean ± SD; two series of experiments, each with N = 10–12 individuals). Color code indicates amplitude of movement, with warm colors representing high levels. Quantification of the leg movements in the ROIs as a function of time (F and G) (mean ± SEM; two independent series of experiments, each with N = 10–12 individuals). Non-parametric Mann-Whitney U test was performed to compare groups; ^∗∗∗^p < 0.001 and ^∗∗∗∗^p < 0.001. A.U., arbitrary units. See also Figures S2 and S4.

Video S1. Self-Righting Movement in Wild-Type *Drosophila* Adults, Related to Figures 1 and 2 and STAR Methods

Video S2. Self-Righting Movement in *miR-iab4/8* Mutant *Drosophila* Adults, Related to Figures 1 and 2 and STAR Methods

Comparison of SR time across different developmental stages reveals that the older the animal, the faster it can self-right (Figure S1C). In regard to the specificity of the effects of *miR-iab4/8* on adult SR, analysis of free-walking behavior in adult flies (STAR Methods) shows that the mutation of the *miR-iab4/8* locus does not impair broad aspects of exploratory locomotion in adult flies (Figure S2). This is important and indicates that the absence of the *miR-iab4/8* system does not lead to a generalized motor-deficient phenotype. However, previous work [32] showed that *miR-iab4/8* mutants displayed posture control defects during mating, suggesting that *miR-iab4/8* may function in other posture control systems in addition to SR. Alternatively, these different posture control systems may share some of the same neural substrate upon which *miR-iab4/*8 exerts its biological role.

The *miR-iab4/8* locus encodes two distinct miRNA molecules: *miR-iab4* [34] and *miR-iab8* [32, 35, 36], each produced from pri-miRNA transcription of opposite DNA strands (Figure S3A). To tease apart the individual contributions of *miR-iab4* and *miR-iab8* toward adult SR, we performed a series of genetic complementation tests using a collection of chromosomal variants bearing different breakpoints (Figure S3B) that specifically disabled the synthesis of *miR-iab4* (*iab-3*^*277*^) or *miR-iab8* (*iab-7*^*MX2*^) [32, 37] precursor pri-miRNAs. By placing these rearrangements in combination with the *miR-iab4/8* mutation (*ΔmiR*), we determined that *miR-iab4* (and not *miR-iab8*) is responsible for the effects on the adult SR response (Figure S3C).

Behavioral observation of the SR routine in the adult shows that legs perform a key role during the SR response (Figure 2A; Videos S1 and S2), allowing the animal to swiftly grab the substrate and use this point of contact to flip its body into the right-side up position. The halteres, important mechanosensory organs that control body maneuvers in flight [38, 39, 40, 41, 42], may also contribute to the control of body maneuvers underlying the SR response. However, when SR tests as described above (Figure 1C) were conducted in flies with ablated halteres, we observed no effects on the time required to complete the SR response as compared to controls (Figure S4A). Next, we asked which pair of legs derived from the pro- (T1), meso- (T2), or meta- (T3) thoracic segments contributed to the control of SR. We performed a series of ablation experiments in which we surgically removed T1, T2, or T3 legs from wild-type individuals and assessed their performance in SR tests (Figure S4B). These experiments showed that the activities of T1 and T3 legs contribute to normal SR, whereas removal of T2 legs had no detectable effects.

We then sought to establish whether leg movement showed any anomalies in *miR-iab4/8* mutant flies when compared with wild-type specimens. Quantification of leg movement in glued (immobilized) upside-down flies (Figure 2B) showed that in *ΔmiR* mutant flies, legs displayed a reduction in the amplitude of leg movement (Figures 2C and 2D), their dynamic range (Figure 2E), and fraction of time spent moving legs (Figures 2F and 2G). These characteristics led to a decrease in overall activity levels compared to those observed in normal flies (Figures 2C–2G). This observation suggests that the impact of *miR-iab4* on the SR response is mediated—at least in part—through specific effects in the levels of movement of the legs in upside-down flies (NB: a general decrease in leg activity should affect walking behavior, but we detect no statistically significant support for this; Figure S2).

**Figure 3:**
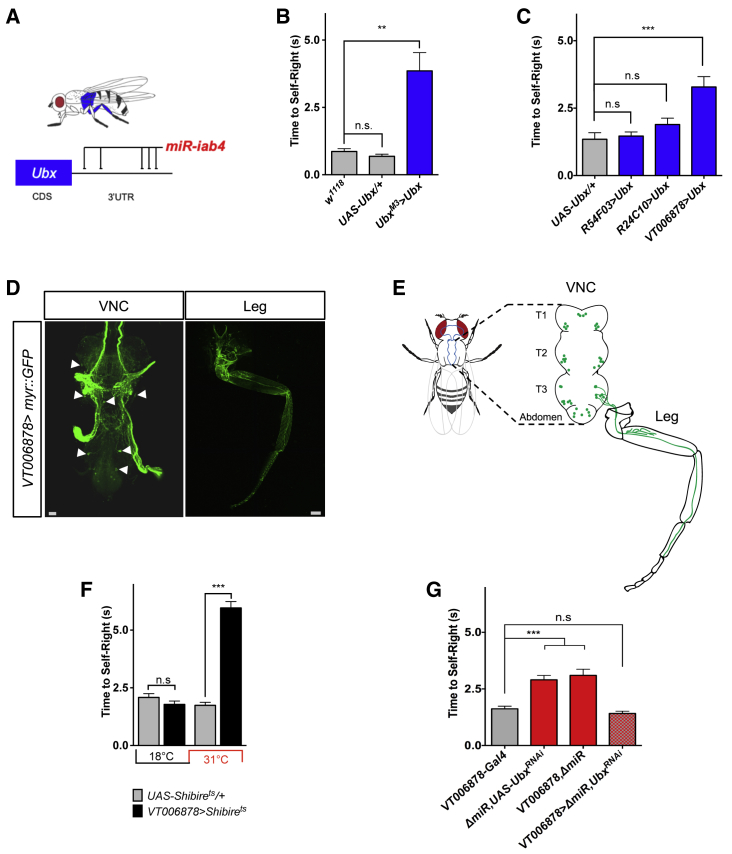
miRNA-Dependent *Ubx* Regulation in Ventral *VT006878*/ventral lin15 Motor Neurons (NB2-3/lin15) Underlies Adult SR Behavior Roles of specific motor neuron subpopulations in SR behavior. (A) The *Hox* gene *Ubx* is expressed in the third thoracic region (blue); previous work (see main text) showed that *miR-iab4* regulates *Ubx* expression via specific target sites in *Ubx* 3′UTR sequences. (B) Quantification of SR behavior in adult flies overexpressing *Ubx* within its natural expression domain (*Ubx*^M3^>*Ubx*: *w*; UAS-*Ubx*/+; *Ubx*^M3^-GAL4/+) shows that upregulation of *Ubx* is sufficient to cause an adult SR defect (mean ± SEM; n = 19–25). (C) *Ubx* overexpression in the *VT006878*/lin15 motor neurons innervating T3 legs phenocopies SR abnormal response (*VT006878*>*Ubx*: *w*; *UAS-Ubx*/+; *VT006878-GAL4*/+) (mean ± SEM; three series, each with N = 13–15 flies). (D) Confocal images of *VT006878 > Myr::GFP* (*w*; *UAS-myr::GFP*/+; *VT006878-GAL4*/+) in the VNC (left) and leg (right); arrows show cell bodies. The projection is displayed to show a maximum of cell bodies by preventing neurite projections. (E) Diagram describing the pattern of *VT006878*-Gal4 expression in the adult VNC and T3 leg. (F) Blocking neural activity in *VT006878* neurons (*VT006878*>Shibire^ts^: *w*; +; *VT006878-GAL4*,+/+,*UAS-Shibire*^*ts*^) leads to defects in adult SR response (mean ± SEM; N = 57–64 flies). (G) In *ΔmiR* adult flies, RNAi-mediated decrease of *Ubx* expression within the *VT006878* domain rescues the SR phenotype (mean ± SEM; N = 41 flies). *VT006878*, *ΔmiR* (*w*; +; *VT006878-Gal4*,*ΔmiR/+,ΔmiR*), *ΔmiR,UAS-Ubx*^*RNAi*^ (*w*; +; +,*ΔmiR/*UAS-*Ubx*^*RNAi*^*,ΔmiR*), *VT006878*>*ΔmiR,Ubx*^*RNAi*^ (*w*; +; *VT006878-Gal4*,*ΔmiR/*UAS-*Ubx*^*RNAi*^*,ΔmiR*). (NB: Experiments in adult flies were conducted on wingless specimens; see STAR Methods and Figure 1 legend.) Scale bars for anatomic images in (D), 10 μm. Non-parametric Mann-Whitney U (B, C, and F) and one-way ANOVA with the post hoc Tukey-Kramer (G) tests were performed to compare treatments; p > 0.05 (non-significant; n.s.), ^∗^p < 0.05, and ^∗∗∗^p < 0.001. See also Figures S5 and S6.

To explore the molecular basis underlying *miR-iab4* effects on adult SR, we considered the hypothesis that *miR-iab4* exerts its effects on adult SR via the same molecular target established in the larva, the *Hox* gene *Ubx* [21] (Figure 3A). Previous work in our laboratory [21, 43, 44] and elsewhere [34, 35, 36] determined that *miR-iab4* interacts with *Ubx* transcripts in a wide range of cell types through a series of specific miRNA target sites located in the *Ubx* 3′UTR. In addition to being an *miR-iab4* target, *Ubx* plays a key developmental role in allocating morphological specificity to the third thoracic ganglion and segment (T3) [16, 17, 18, 19], including effects on the morphological patterning of the T3 leg (Figure 3A) [45]. To test the model that *miR-iab4* represses *Ubx* to allow for normal adult SR response, we increased the expression levels of *Ubx* within its natural transcriptional domain in normal flies seeking to emulate the de-repression effects caused by miRNA removal. The results of this experiment (Figure 3B) show that an increase of *Ubx* levels phenocopies the effects of the *miR-iab4/8* mutation on adult SR response, suggesting that the expression levels of *Ubx* are important for normal behavior.

Taking into consideration (1) that mutation of the *miR-iab4/8* locus disrupts the SR response, (2) that legs play a key role in SR, and (3) that modulation in the levels of the *miR-iab4* target *Ubx* within its transcriptional domain had significant impact on SR, we decided to explore the cellular basis underlying SR control by testing the model that modulation of *Ubx* in leg motor neurons—the direct effectors of leg activity—may play a role in the adult SR response. For this, we artificially upregulated *Ubx* in different neuronal assemblies known to innervate the *Drosophila* leg [46, 47, 48] using the available lineage-specific leg motor neuron GAL4 drivers *VT006878-GAL4* (NB2-3/lin15) and *R24C10-Gal4* (NB5-7/lin20) (Figures 3C–3E and S5A). These experiments showed that upregulation of *Ubx* within the domain demarked by the *VT006878-Gal4* [49] was sufficient to cause a statistically significant increase in the time that individual flies take to complete the SR response (Figures 3C and S5B), whereas the other motor neuronal driver had no effect. Given that, in the larva, induction of *Ubx* using the *R54F03*-Gal4 is sufficient to trigger SR defects equivalent to those observed in miRNA mutants [21], we tested whether the *R54F03-Gal4* driver was active in the adult, and having confirmed this (Figures S5A and S5C–S5E), we investigated whether *R54F03*-driven expression of *Ubx* had any effects on adult SR. The results of this experiment show that this is not the case (Figures 3C and S5B), suggesting that the miRNA-Hox system operates in distinct cellular foci at different developmental stages.

To further explore the roles of *VT006878* neurons (Figures 3C–3E and S5B) in regard to adult SR, we conducted a neuronal inhibition experiment through expression of a temperature-sensitive allele of *shibire* [50]; this treatment has pervasive effects on the timing of the SR response (Figure 3F), indicating that normal activity of the neurons labeled by this line is essential for a normal SR response. Detailed analysis of *VT006878* expression suggests that this driver is not only active in leg motor neurons but also shows signal in wing and haltere sensory axons (Figure 3D), making it plausible that wings and/or halteres may play a role in adult SR. However, two manipulations indicate that the effect we observed by overexpression of *Ubx* in the *VT006878-Gal4* line emerges from a role in leg motor neurons. First, all adult flies had surgically removed wings in our SR behavioral paradigm, making it unlikely that mechanosensory signals from these appendages are key contributors to this behavior. Second, as mentioned above, ablation of halteres resulted in no apparent effect in the time wild-type flies took to complete an SR response (Figure S4A).

Upregulation of *Ubx* using *VT006878-Gal4* is expected to increase *Ubx* levels in all three thoracic segments (T1–T3) and scattered neurons in the brain (Figures 3C and S5B) [49], making it unclear whether ectopic *Ubx* expression in the brain per se might be the cause of the SR defects observed in treated adults. To test this possibility, we constrained the expression pattern of *VT006878 > Ubx* to the brain only, using the ventral nerve cord (VNC)-specific tool *teashirt-Gal80* (Tsh-Gal80) to repress GAL4 activity in the VNC (Figure S6A). Upregulation of *Ubx* within circuits in the brain (plus *VT006878*-driven areas in the wing and haltere sensory axons) has no effect on the timing of SR in adults (Figure S6B), indicating that (1) an increase of *Ubx* within the *VT006878* domain in the brain is insufficient to cause an adult SR phenotype, and (2) *Ubx* upregulation within the thoracic *VT006878* domain is indeed responsible for the triggering of SR defects in the adult. Furthermore, decapitated flies—which are commonly used to probe the role of the brain in a wide range of behaviors [51]—are able to self-right (Figure S6C), indicating that the brain is not essential for this behavior.

To explore the functional implications of *Ubx* expression control within the *VT006878* domain, we performed a series of RNAi knockdown experiments aimed at reducing the levels of *Ubx* expression specifically in *VT006878* motor neurons in *ΔmiR* adult mutants. The results of this experiment (Figure 3G) demonstrate that *Ubx*^*RNAi*^ expression driven by *VT006878-Gal4* rescues the SR phenotype in *ΔmiR* adult flies, suggesting that levels of Ubx protein in these neurons might be critical for normal SR (see below).

**Figure 4:**
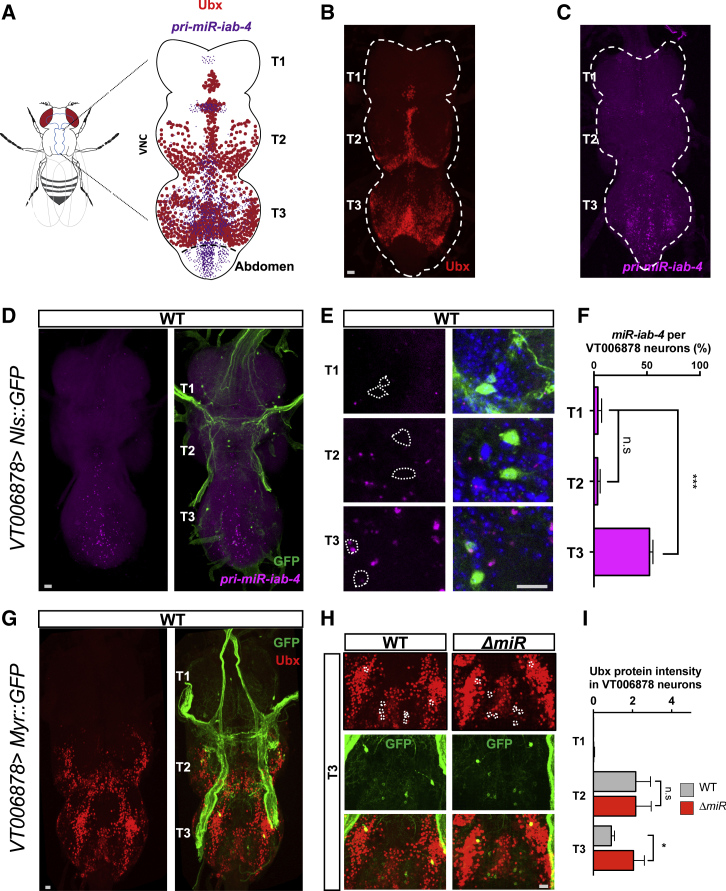
Expression Analysis of Ubx Protein and *miR-iab-4* in the Adult VNC (A) Schematic diagram of the adult VNC showing areas of *miR*-*iab*-*4* (magenta) and Ubx protein (red) expression. (B) Expression of Ubx protein within the VNC shows highest signal in the T3 ganglion, but signal is also detectable in T2 and a much lower level in T1. (C) *miR-iab-4* expression in the VNC shows highest level of expression in the T3 ganglion. (D) *miR-iab-4* expression profile in *VT006878*-positive neurons. *VT006878* neurons labeled by GFP (*VT006878*>*Nls*::*GFP*: *w*; UAS-*Nls*::*GFP*/+; *VT006878-Gal4/+*). (E and F) Quantification of *miR-iab4* signal (magenta) within the *VT006878* domain (green) along different ganglia of the VNC shows a significant increase of miRNA signal in the T3 ganglion (blue, DAPI) (mean ± SEM; n = 7 VNCs). (G) Expression of Ubx protein (red) is detected within the *VT006878* domain labeled by GFP (green). *VT006878* neurons labeled by GFP (*VT006878*>*Myr*::*GFP*: *w*; *UAS*-*Myr::GFP*/+;*VT006878-Gal4/+*). (H) Expression pattern of Ubx protein within the *VT006878* domain in the T3 region of the VNC in WT (*w*; *UAS*-*Myr::GFP*/+; *VT006878-Gal4/+*) and miRNA mutants (*ΔmiR*: *w*; *UAS*-*Myr::GFP*/+; *VT006878-Gal4*, *ΔmiR/+, ΔmiR*). A significant increase in Ubx protein expression is observed in the T3 ganglion of mutant adult flies; in contrast, comparison of normal and miRNA mutant flies shows no significant differences in Ubx expression in the T2 ganglion (mean ± SEM; n = 7–13 VNCs). Scale bars for anatomic images, 10 μm. Non-parametric Mann-Whitney U and one-way ANOVA with the post hoc Tukey-Kramer tests were performed to compare treatments; p > 0.05 (not significant; n.s.), ^∗^p < 0.05, and ^∗∗∗^p < 0.001. See also Figure S7.

Immunolabelling experiments show that Ubx protein is expressed in subsets of adult neurons within the T1–T3 ganglia, with a larger population observed within the T3 segment of the VNC (Figures 4A and 4B). RNA *in situ* hybridizations show that *miR-iab4* is highly expressed in the T3 ganglion of the VNC (Figures 4A and 4C) and that both Ubx and *miR-iab4* are expressed within the *VT006878* domain (Figures 4D–4I). In miRNA mutants, Ubx expression is significantly increased in the T3 segment of the VNC, but not in T2 (Figures 4H, 4I, and S7), in agreement with the idea that increase of *Ubx* expression (de-repression) within the *VT006878* domain in T3 leads to SR defects in the adult. A prediction that emerges from this idea is that artificial reduction of *Ubx* in *ΔmiR* mutants, specifically confined to the *VT006878* domain, should ameliorate (or even rescue) the SR phenotype observed in adult mutants. In line with this prediction, as mentioned above, RNAi-mediated reduction of Ubx driven by *VT006878-Gal4* rescues the SR phenotype in adult flies (Figure 3G).

**Figure 5:**
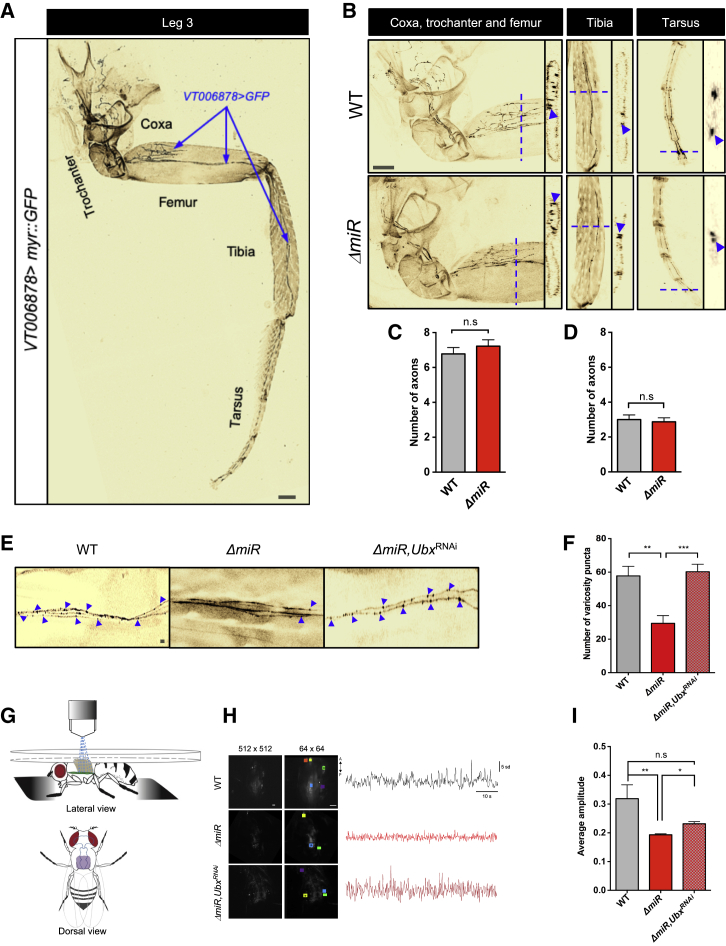
Effects of miRNA Mutation on the Morphology and Function of *VT006878*/ventral lin15 Motor Neurons (NB2-3/lin15) (A) Image of hind (T3) leg showing *VT006878*-positive neuronal projections labeled by GFP (*VT006878*>*Myr::GFP*). *VT006878* neurons innervate the coxa, trochanter, femur, tibia, and tarsus segments. (B) Projections of *VT006878* neurons into the coxa, trochanter, femur (left), tibia (middle), and tarsus (right) of wild-type (WT; *w*; *UAS-Myr::GFP*/+; *VT006878-Gal4*/+) and miRNA mutants (*ΔmiR*; *w*; *UAS*-*MyrGFP*/+; *VT006878-Gal4*, *ΔmiR/+, ΔmiR*) specimens show no significant differences across genotypes. For each segment: medial view, left; cross-section, right (mean ± SEM; N = 9 flies per genotype). (C and D) Quantification of *VT006878* projections in the segments shows no significant effects of the miRNA system on *VT006878*/lin15 morphology (dashed line indicates the plane of a cross-section shown at the right of each segment figures; arrowheads highlight motor neuron projections analyzed) (mean ± SEM; N = 8 flies per genotype). (E and F) Varicosity puncta of *VT006878* projections in WT and *ΔmiR* femur. Note the significant reduction in puncta observed in miRNA mutants and the effect caused by *Ubx* RNAi (*ΔmiR,Ubx*^*RNAi*^: *w*; *UAS*-*myr::GFP*/+; *VT006878-Gal4*, *ΔmiR/* UAS-*Ubx*^*RNAi*^*, ΔmiR*) treatment within the *VT006878* domain in miRNA mutants, which rescues the normal number of puncta as observed in WT samples (mean ± SEM; N = 10–12 flies per genotype). (G) Schematic representation of the preparation used for calcium activity recordings (top) and the scanned T3 segment (bottom). (H and I) Calcium activity of *VT006878* neuron somata and projections within VNC. Representative image for high-resolution morphology (512 × 512) and activity (64 × 64) scans (colored ROIs are detected semi-automatically by Igor software from calcium activity traces in those areas) (left) and an example of calcium activity traces within an ROI (i.e., ROI labeled by a star) reported by GCAMP6m in time indicated by standard normalized fluorescence (SD) (right) (H). Average amplitude, representing area under the curve of the time series, averaged over ROIs (I) of WT (*w*; *UAS*-*GCAMP6m*/+; *VT006878*-*Gal4*/+), miRNA mutants (*ΔmiR*; *w*; *UAS*-*GCAMP6m*/+; *VT006878-Gal4*, *ΔmiR/ +, ΔmiR*), and rescue (*ΔmiR,Ubx*^*RNAi*^: *w*; *UAS*-*GCAMP6m*/+; *VT006878-Gal4*, *ΔmiR/* UAS-*Ubx*^*RNAi*^*, ΔmiR*) flies (mean ± SEM; N = 5–6 flies per genotype). Non-parametric Mann-Whitney U (C and D) and one-way ANOVA with the post hoc Tukey-Kramer (F and I) tests were performed to compare treatments; p > 0.05 (non-significant; n.s.), ^∗^p < 0.05, ^∗∗^p < 0.01, and ^∗∗∗^p < 0.001. Scale bars for anatomic images, 10 μm.

Detailed anatomical examination of T3 *VT006878* leg motor neurons (also known as NB2-3/lin15 or *ventral lineage 15 motor neurons* [49]) in wild-type and *ΔmiR* specimens showed no detectable differences in axonal projections or morphologies (Figures 5A–5D), suggesting that—as observed in the larva [21]—the miRNA under study might have effects on neuronal function, rather than on neuronal morphology. Indeed, quantification of varicosities (a known indicator of neuronal activity [52, 53]) at the junction of *VT006878* neurons with the muscle system of the third leg reveals a statistically significant reduction in varicosities in the *ΔmiR* samples (Figures 5E and 5F) in line with the model that absence of the miRNA leads to diminished levels of neural activity. Remarkably, in *ΔmiR* mutants, RNAi-mediated reduction of *Ubx* expression in the *VT006878* neurons rescues the normal number of varicosities, strongly indicating a role of *Ubx* in the formation of active contact points between the neuronal and muscle systems. Furthermore, multiphoton microscopy analysis (Figure 5G) of genetically encoded calcium reporters (GCaMP6m) [54] specifically expressed in the *VT006878* motor neurons shows an overall reduction of spontaneous neural activity in *ΔmiR* samples in T3 when compared to wild-type (Figures 5H and 5I). Remarkably, as observed in the varicosity analysis, a reduction of *Ubx* mediated by *VT006878*>*UbxRNAi* significantly increases the levels of neural activity in *VT006878* neurons (Figures 5H and 5I) partially recovering activity levels. Altogether, our data suggest that *miR-iab4* represses *Ubx* within the *VT006878* motor neuron domain in T3, ensuring the normal neural functions that underlie the adult SR response.

**Figure 6:**
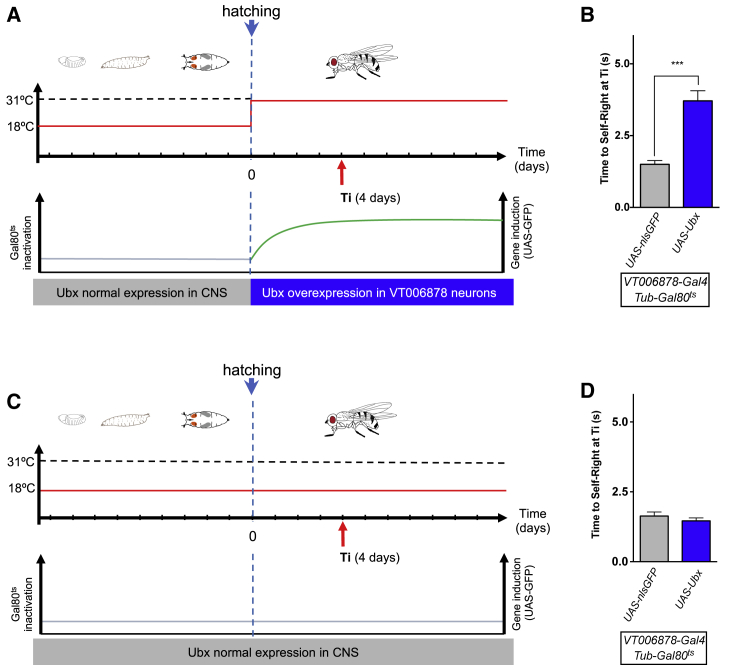
Conditional Increase of *Ubx* Expression after Development Is Completed Is Sufficient to Alter Adult Behavior (A) Conditional expression experiment in which Ubx protein is upregulated only once development has been completed. Graphic representation of *Gal4* and *Gal80* activities over developmental time. NB: At 18°C, *Gal80*^*ts*^ represses Gal4 activity; at 31°C, the *Gal80*^*ts*^ role is inactivated, allowing for *VT006878*-*Gal4*-mediated induction of Ubx (green) in ventral lin15 motor neurons. Maximal induction is achieved approximately 4 days after eclosion (Ti). (B) SR behavior test (STAR Methods) performed at Ti reveals that post-developmental induction of Ubx in *VT006878* neurons *Tub-Gal80*^*ts*^; *VT006878* > *Ubx* (*w*; *UAS-Ubx*/+; *Tub-Gal80*^ts^,+/ +, *VT006878-Gal4*) (blue) is sufficient to cause SR defects in comparison to control line *Tub-Gal80*^*ts*^*;VT006878* > *Nls::GFP* (*w*; *UAS- Nls*::*GFP* /+; *Tub-Gal80*^ts^,+/ +, *VT006878-Gal4*) (mean ± SEM; N = 19–25 flies). A non-parametric Mann-Whitney U test was performed to compare treatments; ^∗∗∗^p < 0.001. (C and D) Control treatment for the conditional expression of *Ubx* in adult *Drosophila*. Graphic representation of *Gal4* and *Gal80* activities over developmental time (C). At 18°C, Gal80^ts^ represses Gal4 activity, thus blocking *VT006878*-*Gal4*-mediated induction of *Ubx* in *VT006878*/ventral lin15 neurons. Under Gal80-mediated repression, there is no induction of *Ubx* expression in *VT006878* cells and no statistically significant changes in SR times are observed when comparing the experimental line *Tub-Gal80*^*ts*^*;VT006878;* > *Ubx* (blue) with the control line *VT006878;Tub-Gal80*^*ts*^>*Nls::GFP* (gray) (mean ± SEM; N = 19–25 flies) (D). A non-parametric Mann-Whitney U test was performed to compare treatments; ^∗∗∗^p < 0.001. (NB: Experiments in adult flies were conducted on wingless specimens; see STAR Methods and Figure 1 legend.)

Lastly, we sought to determine whether the effects of the miRNA on adult SR behavior emerge from a progressive developmental function of the miRNA, or rather are the consequence of the activity of the miRNA on the physiology of the *VT006878* motor neurons in the adult. For this, we performed a conditional expression experiment to mimic the de-repression effect of absence of *miR-iab4* on its target *Ubx* during specific developmental intervals. In the experiment, we maintained normal expression of *Ubx* in the *VT006878* domain during the full developmental process that spans from embryo to adulthood, increasing *Ubx* expression only after adult eclosion (Figures 6A and 6C). Our data show that an increase in *Ubx* expression, exclusively delivered in the adult, is *sufficient* to induce SR defects compared to controls (Figures 6B and 6D), revealing a post-developmental role of the *Hox* genes in the control of neural function in the fully formed organism.

## Discussion

Our work reveals that functionally equivalent adaptive movements performed by organisms with distinct biomechanical, morphological, and neural structures can rely on a simple genetic module involving an miRNA and a *Hox* gene. The findings open up the possibility that other functionally equivalent behaviors that manifest in different developmental stages within the life cycle of an organism may also rely on common genetic modules.

**Figure 7:**
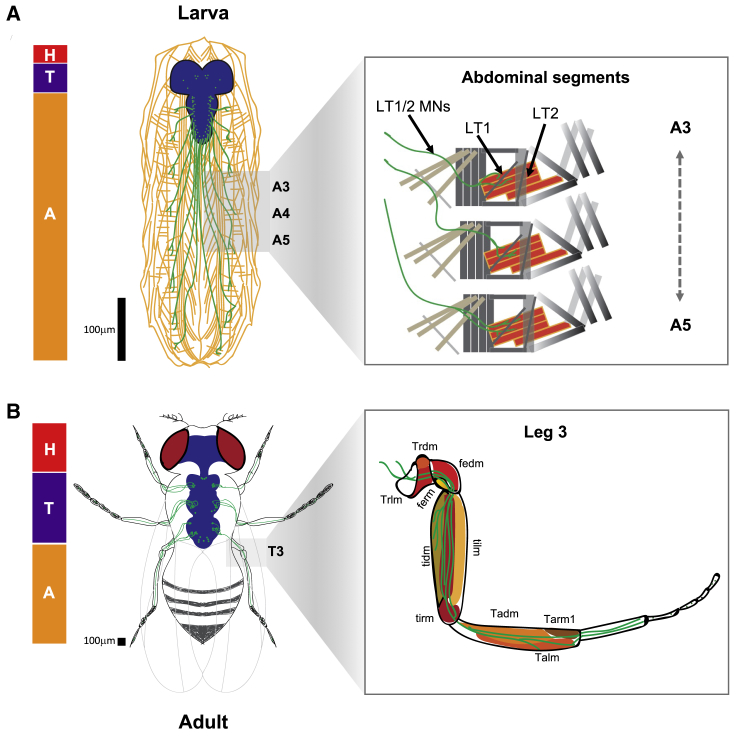
Concept Diagram Comparing the Current Understanding of the Neural Basis of Self-Righting Behavior in the *Drosophila* Larva and Adult (A) Tracings of the larval body wall muscles and lateral transverse muscles 1/2 motor neurons (LT1/2 MNs) [21], projections (green) in the abdominal segments (left), and the illustration of body wall muscles innervated by the motor neurons located in an abdominal hemisegment (A_3_–A_5_). In the larva, LT1/2 motor neurons innervate LT1 and LT2 muscles in the body wall; previous work showed that *miR-iab4* and *Ubx* play a particularly important role in the function of these neurons in abdominal segments A3 to A5. (B) In the adult, evidence presented in this study indicates that the *miR-iab4*::*Ubx* module is important for normal activity of the *VT006878*/ ventral lin15 motor neurons in the third thoracic segment. These motor neurons extend complex projections into different leg muscles including the coxa, trochanter, femur, and tibia muscles. The muscles are labeled as described previously [55]: Talm, tarsus levator muscle; tadm, tarsus depressor muscle; tarm, tarsus reductor muscle; tilm, tibia levator muscle; tidm, tibia depressor muscle; tirm, tibia reductor muscle; fedm, femur depressor muscle; ferm, femur reductor muscle; trlm, trochanter levator muscle; trdm, trochanter depressor muscle. The bars to the left of (A) and (B) represent the anatomical size of the three main segments: head (H, red), thorax (T, magenta), and abdomen (A, orange).

Our data suggest that the *Drosophila* LT1/2 motor neurons, which are essential for normal SR control in the larva [21], play no evident role in the adult (Figures 3C and S5B). A possible interpretation of these observations is that the miRNA-Hox system might be re-deployed in different elements of the neural network underlying SR at distinct developmental stages. While we are currently using a connectomics approach to map the neural circuitry underlying SR (unpublished data), no full understanding of the circuit is available at present, making it difficult to establish a one-to-one cellular comparison across the larval and adult SR circuits, or draw any categorical conclusions regarding the relation between the cellular foci required for SR at different stages. However, there is strong indication that neurons located in different tagma are involved in larval and adult SR. For instance, in the first instar larva, gene expression and neural activity data show that LT1/2-MNs within abdominal segments A3 to A5 play a key role (Figure 7A) [21], while in the adult, our gene expression data show that thoracic neurons (e.g., *VT006878*-MNs), in particular those in the third thoracic ganglion, are crucial for normal SR (Figure 7B). Furthermore, *VT006878*-driven expression of *Ubx* in the larva does not cause any detectable SR defects at that stage (Figures S6D and S6E). These observations lend support to the idea that the *miR-iab4::Ubx* module may have been co-opted by distinct cellular components of the nervous system acting in different tagma of the larva and adult.

The study also reveals what is—to our best knowledge—the first case of a post-developmental role of the *Hox* genes with impact on neural physiology and behavior.

Several earlier investigations have reported roles for the *Hox* genes in adult “non-neural” tissues including the *Drosophila* heart [56], midgut [57], and muscle [58], focusing on the developmental roles of the *Hox* system. Indeed, in humans, *Hox* gene de-regulation is considered a hallmark for several types of cancer [59, 60, 61, 62], suggesting an important role of these genes in adult cell biology with clinical implications. Within the nervous system, although many studies have previously revealed roles for the *Hox* genes in neuronal patterning, survival, and differentiation, including axonal branching and terminal and post-embryonic neural differentiation [63, 64, 65, 66, 67, 68, 69], to our best knowledge, the roles of these key developmental factors have never been linked to neurophysiological regulation or differential behaviors in fully formed organisms. Indeed, our analysis of *Ubx* expression in the adult CNS surprisingly emerges among the first characterizations of the expression domain of *Ubx* within the *Drosophila* adult nervous system [65].

Our results reveal that modulation of *Hox* expression plays an essential role in the control of normal adult behavior and that changes in *Hox* gene inputs can regulate the physiology of neurons (Figures 5E–5I). These findings imply that *Hox* expression levels must be kept in the vicinity of a “set-point” to drive normal neural physiology and behavioral control. We are currently testing this notion and extending this work by systematically mapping the neurophysiological and behavioral roles of all the *Hox* genes in the adult fly. The fact that the *Hox* genes can contribute to both the neuro-developmental process and the physiological setting of the nervous system suggests that an extreme interpretation of the "Brenner paradigm" (see Introduction) in the form of a categorical classification of genes according to roles in either the construction or the physiological specialization of the nervous system might hamper, rather than benefit, the understanding of the genetic specification of nervous systems.

Interestingly, *Ubx* and its regulatory miRNA *miR-iab4* are both located within the *Bithorax* complex, only ∼120 kb apart from one another; therefore, the integrity of this small segment of the chromosome is responsible not only for normal *Drosophila* development, but also for the adequate neurophysiological regulation underlying the coordination of adaptive movements displayed by different morphs of the fly. More broadly, our observations provide a new example of the remarkable capacity of the genome as a multilayer information storage system able to guide both the formation and function of a complex organism at different points during its life cycle.

Based on the wide evolutionary conservation on the *Hox* gene system and the key roles played by these genes in the nervous systems of animals as different as insects and mammals [17, 19], we propose that similarly simple and compact genetic modules including *Hox* genes and their regulatory miRNAs may conform part of the molecular circuitry underlying movement control in other species, including humans.

## STAR★Methods

### Key Resources Table

**Table:** 

REAGENT or RESOURCE	SOURCE	IDENTIFIER
**Antibodies**		

mouse monoclonal anti-Ubx	DSHB	Ubx FP3.38 DSHB Cat# Ubx FP3.38; RRID: AB_10805300
chicken anti-GFP	Abacam	Ab13970 Abcam Cat# ab13970; RRID: AB_300798
anti-mouse Alexa Fluor 555	Thermo Fisher	A21202 Thermo Fisher Scientific Cat# A-21202; RRID: AB_141607
anti-chicken Alexa Fluor 488	Jackson Immunoresearch	703-545-155 Jackson ImmunoResearch Labs Cat# 703-545-155; RRID: AB_2340375

**Chemicals, Peptides, and Recombinant Proteins**		

37% formaldehyde	Sigma-Aldrich	F8775
Glycerol	Fisher BioReagents	BP229-1
Triton X-100	Sigma-Aldrich	T8787
*miR-iab4* probes	Stellaris, Biosearch Technologies	https://www.biosearchtech.com/support/education/stellaris-rna-fish
UV-activated glue	BONDIC	N/A
Sigmacote	Sigma-Aldrich	Cat# SL2

**Experimental Models: Organisms/Strains**		

*VT006878-Gal4*	VDRC	ID200694
*R54F03-Gal4*	BDSC	#39078
*R24C10-Gal4*	BDSC	#49075
*Tubulin*-*Gal80*^*ts*^	BDSC	#7018
*ΔmiR-iab4*/*iab8*	[32]	Gift from Welcome Bender
*Iab*-3^277^	[37]	Gift from Ernesto Sánchez-Herrero
*Iab*-5^105^	[37]	Gift from Ernesto Sánchez-Herrero
*Iab*-*7*^*MX2*^	[37]	Gift from Ernesto Sánchez-Herrero
*UbxM3*-*Gal4*	[70]	Gift from Ernesto Sánchez-Herrero
*UAS*-*UbxIa*	BDSC [71],	#911
*UAS*-*UbxRNAi*	BDSC	#31913
*UAS*-*Myr*::*GFP*	BDSC [72],	#32198
*Tsh*-*Gal80*	[73]	Julie Simpson lab
*UAS*-*Nls*::*GFP*	BDSC	#4775
*UAS-GCaMP6m*	BDSC [54],	#42748
UAS-*shibire*^*ts*^	BDSC [50],	#44222
G-trace	BDSC [74],	#28281

**Software and Algorithms**		

Igor Pro 6.3 and 8	WaveMetrics	N/A
Fiji	NIH	http://fiji.sc/
MATLAB	MathWorks	https://ch.mathworks.com/products/matlab.html
Prism	GraphPad Software	https://www.graphpad.com/
Ctrax	[75]	http://ctrax.sourceforge.net/install.html
Visual Studio	Microsoft	N/A
Bonsai	Bonsai Reference [76]	http://www.kampff-lab.org/bonsai

**Other**		

Leica TCS SP8 microscope	Leica Microsystems	https://www.leica-microsystems.com/products/confocal-microscopes/details/product/leica-tcs-sp8/
Flea FL3-U3-32S2M camera	Point Grey	N/A
M1214-MP2 lens	Computar	N/A
Bonito CL-400B camera	Allied Vision	N/A
EX2C	Computar	N/A
PCIe-1433	National Instruments	N/A

### Lead Contact and Materials Availability

Further information and requests for resources and reagents should be directed to and will be fulfilled by the Lead Contact, Claudio R. Alonso (c.alonso@sussex.ac.uk). This study did not generate new unique reagents.

### Experimental Model and Subject Details

*Drosophila melanogaster* were reared on standard medium (water, agar, cornmeal, molasses, yeast, nipagin, propionic acid) in standard tubes or bottles, and maintained at 25°C in a temperature-controlled incubator at 50% humidity with a 12 h/12 h cycle of alternating light and dark.

Four-day-old females or males were used with their wings cut under cold anesthesia one day prior to the self-righting experiment. Wild-type, w^1118^ flies served as control in all experiments or were used to generate heterozygous flies. For silencing of *VT006878* neurons or conditional overexpression of *Ubx* mediated by *shibire*^ts1^ or *Tub*-*Gal80*^*ts*^ respectively, eggs, embryos, larvae and pupae were raised at 18°C and the freshly hatched flies were transferred to 31°C. All the experiments were conducted at 25°C.

### Method Details

#### Self-Righting tests

Larval SR behavior was assayed as previously described [21, 29]. For adult SR behavior tests, flies were grown in non-crowed conditions at 25°C. The day before the SR test, the wings of cold-anesthetized 2-to-4-day old flies were surgically removed (clipped). Flies recovered for one day at 25°C. Flies were assayed for SR behavior by being introduced individually into an arena and rolled over with a brush to an “upside-down” position (“legs up”) and the time taken by the fly to return to its normal position (“right-side up”) was recorded (Videos S1 and S2). A maximum of ten minutes was given to the fly to SR. All experiments were done with flies 4-6 days after eclosion and tested at 25°C. Similar results to those observed using this procedure were obtained when measuring SR time in adult flies with intact wings after recovery from CO_2_-induced or cold-induced anesthesia (Figure S1). The absence of halteres showed no effects on SR (Figure S4A). For silencing of *VT006878* neurons, 3- to 4-day-old flies expressing *shi*^ts1^, were incubated for 10 min at the restrictive temperature of 31°C, or at the non-restrictive temperature of 18°C for controls, just before the SR test. SR behavior was assessed within seconds (50 ± 10) after incubation.

#### Walking behavior

Locomotion in single flies (males or females) was assessed in a 58mm diameter circular arena with sloped edges (11° [77]) to restrict walking to the center of the arena. Spontaneous walking behavior was recorded for 15 min from the top using a monochrome Flea FL3-U3-32S2M Point Grey camera with a M1214-MP2 lens (Computar), with a resolution of 1024x1024 pixels at 60 Hz. Acquisition was controlled with a custom-made Bonsai script [76]. To prevent walking on the ceiling, arenas were covered with glass that was pre-coated with Sigmacote (SIGMA-ALDRICH). To automatically track the position of flies in the arena, we used Ctrax [75], a machine-vision algorithm that automatically computes multiple walking relevant parameters, such as speed, orientation, etc. Walking bouts were defined as segments in time when the body moved through space with a minimum speed of 5 mm/s, for at least 500ms. As a measurement of locomotion performance, we calculated the straightness of a walking bout (Figures 2B–2G). Straightness was defined as the mean angular deviation from a line defined between the start and end points of the segment. A straightness of 0 indicated walking along a perfect straight line. Straightness greater than 0 indicated curvilinear trajectories, and the greater the value, the more prominent the deviations were from a straight course.

#### Quantification of leg movements

Flies 3-5 days post eclosion old flies were cold-anesthetized and their thorax were tethered to a glass microscope slides with UV-activated glue (BONDIC). Leg movement was tracked via fast video recordings at a resolution of 800x800 pixels at 200 Hz with a monochrome digital camera (Bonito CL-400B, Allied Vision, with a M1214-MP2 lens and EX2C extender from Computar). The camera was connected to aPCIe-1433 (National Instruments) frame grabber via a custom-made C# script. We used a custom-made MATLAB script to quantify leg activity levels. Regions of interests (ROI) for analysis were automatically drawn based on the center of mass (CM) of the thorax of the fly. Video images were converted into binary values using a threshold, and a time averaged image was calculated. Because the thorax of the fly was glued to a coverslip it was the only part of the fly that remained stationary throughout the video. To isolate pixels corresponding to the fly thorax, we identified those that did not change in intensity for more than 90% of the video. Pixels that did were converted to a background-related pixel. Next, the thorax of the fly and its CM, were extracted using the connected components method. From this binarized image, we calculated the area moment of inertia and aligned and centered the flies to the vertical axis. From the CM, we automatically defined two regions of interest (ROIs), one on each side of the fly that were separated by the width of the fly thorax, and with 700x175 pixel size. For each pixel inside of these ROIs, we extracted the pixel intensity (in A.U) and calculated the change in pixel intensity as a function of time. Leg activity per pixel was classified as 1 if the instantaneous change in pixel intensity was at least 15 pixels per time step, which corresponded to approximately 5% of the total change in pixel intensity. From this, we averaged the change in pixel intensity over the course of the experiment and generated a mean heatmap (over all flies) for wt and *miRiab4/8* flies (Figure 2D). To quantify the average range of activity of each fly (Figure 2D), we used the contour function from MATLAB to identify the regions in the heatmap where the activity level was similar. From these contours, we calculated the average distance of each point to the origin for the range in the azimuth, with the range in elevation being defined as the distance between two diametrically opposite points of the contour. Responses were averaged across ROIs. To quantify the average leg activity for each fly (Figure 2E), we calculated the mean response of both the Left and Right ROIs, normalized by the area of each ROI.

#### Adult leg preparation and mounting

Tissue dissection and mounting were performed as described [78]. Fly legs were dissected with forceps in 0.3% triton in 1x phosphate buffered saline (PBS). Adult legs attached to thoracic segments were fixed in 4% formaldehyde in PBS overnight at 4°C followed by five washes in PBTx for 20 min at room temperature. Next, legs were mounted onto glass slides using 70% glycerol medium for images acquisition using a Leica TCS SP8 confocal microscope.

#### Immunohistochemistry and RNA *in situ* hybridization

Adult brains and ventral nerve cords (VNC) were dissected in 1X PBS. Tissues were then fixed for 1h in 4% formaldehyde in 1X PBS at room temperature. After fixation, brains and VNCs were washed 3 times (30 min per washing) in PBS with 0.3% Triton X-100 (PBTx) and incubated at 4°C overnight in primary antibodies. The following primary antibodies were used: mouse monoclonal anti-Ubx (FP3.38 [79] 1:500 from the Developmental Studies Hybridoma Bank) and chicken anti-GFP (Abacam Probes, 1:3000). The secondary antibodies were anti-mouse Alexa Fluor 555 (Invitrogen Molecular Probes, 1:1000) and anti-chicken Alexa Fluor 488 (Invitrogen Molecular Probes, 1:1000). RNA *in situ* hybridization in adult ventral nerve cords for the precursor RNA transcripts of *miR-iab-4* was performed by designing 48 unique 20nt-probes labeled with Quasar 570 in the Stellaris platform from Biosearch Technologies, and using an adapted version of the protocol by Raj A., et al., 2010 [80]. Images were acquired with a Leica SP8 confocal microscope, processed and analyzed using FIJI ImageJ [81]. The *VT006878* nerve-ending varicosities were quantified by measuring the puncta they covered in *VT006878*-labeled by myr::GFP in leg or VNC.

#### Two-photon calcium imaging

To prepare flies for *in vivo* imaging in VNC (Figure 5G) we adapted existing methods [82, 83]. In brief, a single fly (3–5 days after eclosion) was cold-anesthetized and tethered using UV-curable glue to a piece of aluminum foil that covered a hole in the bottom of a modified polystyrene weighing dish. The fly’s body was positioned such that the dorsal side of the thorax covered the small hole made in the center of the aluminum foil. The dish was then held by blu-tack on a glass microscope slide with ventral side and legs facing the slide. Next, the dish was filled with saline solution and a small hole in the thorax was opened by removing the cuticle and muscles covering the T3 ganglion using sharp forceps and insect pins to avoid damaging nerves. The preparation was positioned under the two-photon microscope (see details below) and spontaneous GCaMP6m activity within *VT006878* neurons was recorded. Composition of saline solution was as used previously [83]: 108 mM NaCl, 5 mM KCl, 2 mM CaCl_2_, 8.2 mM MgCl_2_, 4 mM NaHCO_3_, 1 mM NaH_2_PO_4_, 5 mM trehalose, 10 mM sucrose, 5 mM HEPES pH 7.5. All imaging experiments were performed on a MOM-type two-photon microscope (designed by W. Denk, MPI, Martinsried; purchased from Sutter Instruments/Science Products) equipped with a mode-locked Ti:Sapphire, Chameleon Vision-S laser set at 927nm. Emitted fluorescence was detected with F48x573, AHF/Chroma, and a water immersion objective 20x/1,0 DIC M27 Zeiss was used for image acquisition. For image collection we used custom-made software running under IGOR pro 6 for Windows (Wavemetrics) [84], at 64 × 64 pixel resolution with 7.8 frames/s image sequences for activity scans or 512 × 512 pixel images for high-resolution morphology scans. All data analysis was performed using IGOR Pro 8 (Wavemetrics) and Fiji (NIH). In brief, image sequences were averaged and the ROIs corresponding to *VT006878* neuron activity were defined semi-automatically by custom software [85]. Then, Ca^2+^ traces for each ROI were extracted and baseline correction applied, followed by z-normalization based on the time interval 1-6 s at the beginning of recordings using custom-written routines under IGOR Pro. And the expression of amplitude responses is represented by SD.

### Quantification and Statistical Analysis

Statistical analyses were performed with GraphPad Software Prism using Mann-Whitney U test or one-way ANOVA with the post hoc Tukey-Kramer test. Error bars in figures represent SEM. Significant values in all figures: ^∗^p < 0.05, ^∗∗^p < 0.01, ^∗∗∗^p < 0.001.

### Data and Code Availability

The MATLAB script made to quantify leg activity levels and all other data generated during this study have not been deposited in a public repository but are available from Claudio R. Alonso (c.alonso@sussex.ac.uk) upon request.

## Supplemental Information

Document S1. Figures S1–S7

Document S2. Article plus Supplemental Information
