# Supplementary material for: A Single MicroRNA-Hox Gene Module Controls Equivalent Movements in Biomechanically Distinct Forms of Drosophila
Source: Curr Biol. 2019 Aug 19;29(16):2665–2675.e4. doi: 10.1016/j.cub.2019.06.082 (PMC6710004; doi:10.1016/j.cub.2019.06.082)
Supplement: Document S1. Figures S1–S7 [file mmc1.pdf]

Current Biology, Volume *29*

Supplemental Information

A Single MicroRNA-Hox Gene Module Controls Equivalent Movements in Biomechanically Distinct Forms of *Drosophila*

A. Raouf Issa, João Picao-Osorio, Nuno Rito, M. Eugenia Chiappe, and Claudio R. Alonso

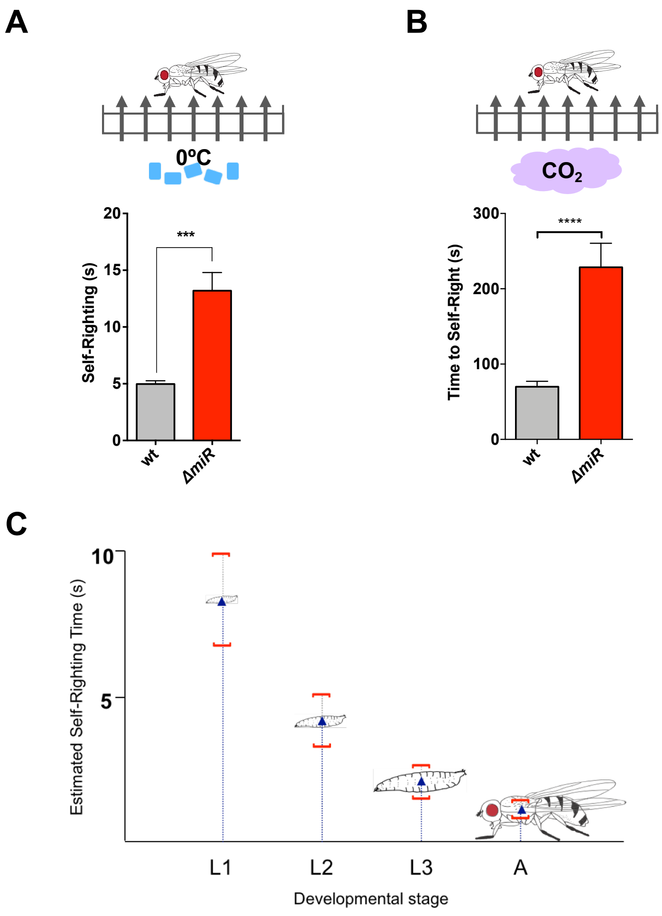

Figure S1. **Mutation of the miRNA locus *miR-iab4/8* leads to self-righting (SR) defects in the *Drosophila* adult. Related to Figure 1. (A** and **B**) Quantification of the ability of wild type (grey) and *miR-iab4/8* mutant flies (red) (*ΔmiR*) to return to normal orientation when turned upside down (self-righting, SR) shows significant effects of the *miR-iab4/8* locus on adult SR when tested in different experimental conditions. Ice anaesthesia (**A**). Prior to the experiment flies were maintained on ice (0ºC) for 10 minutes to allow subject manipulation (mean ± SEM; n = 21-45). CO_2 anaesthesia (**B**). Prior to the experiment flies were anaesthetised by brief exposure to CO_2 (mean ± SEM; n = 29-44). A nonparametric Mann-Whitney U test was performed to compare treatments; P < 0.001 (***). (**C**) Representation of SR time at different developmental stages in Drosophila. SR time was measured in first, second and third instar larvae, i.e. L1, L2, and L3 respectively and in adult flies. Note that in wild type the time required for self-right decreases as the animal moves through the developmental cycle.

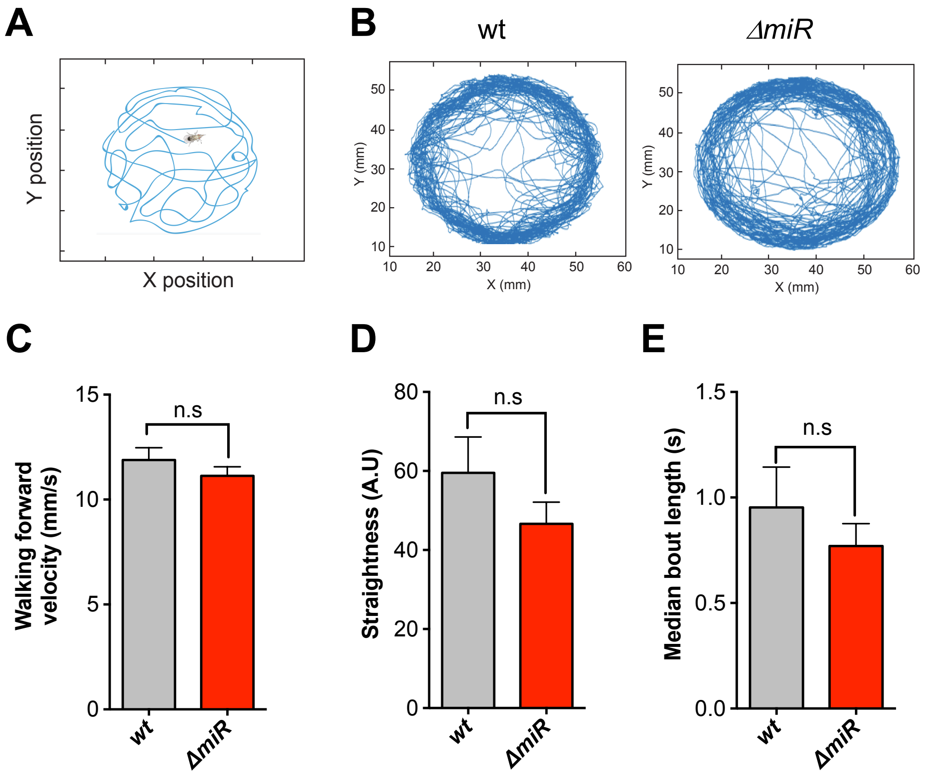

Figure S2. **Genetic removal of *miR-iab4/8* does not affect general locomotion activities in *Drosophila* adults. Related to Figure 2.** (**A**) Concept diagram of Drosophila free walking tracking. (**B**) Path of free walking in wild type and *miR-iab4/8* mutant flies (*ΔmiR*). (**C-E**) Quantification of free walking features across genotypes, walking velocity (**C**), straightness (**D**) and bout length and (**E**) in wild type (grey) and *miR-iab4/8* mutant flies (red) (*ΔmiR*) shows no statistically significant differences among the phenotypes demonstrating that absence of the *miR-iab4/8* system does not lead to a general locomotor deficit in adult flies (mean ± SEM; n = 11-20). A nonparametric Mann-Whitney U test was performed to compare treatments.

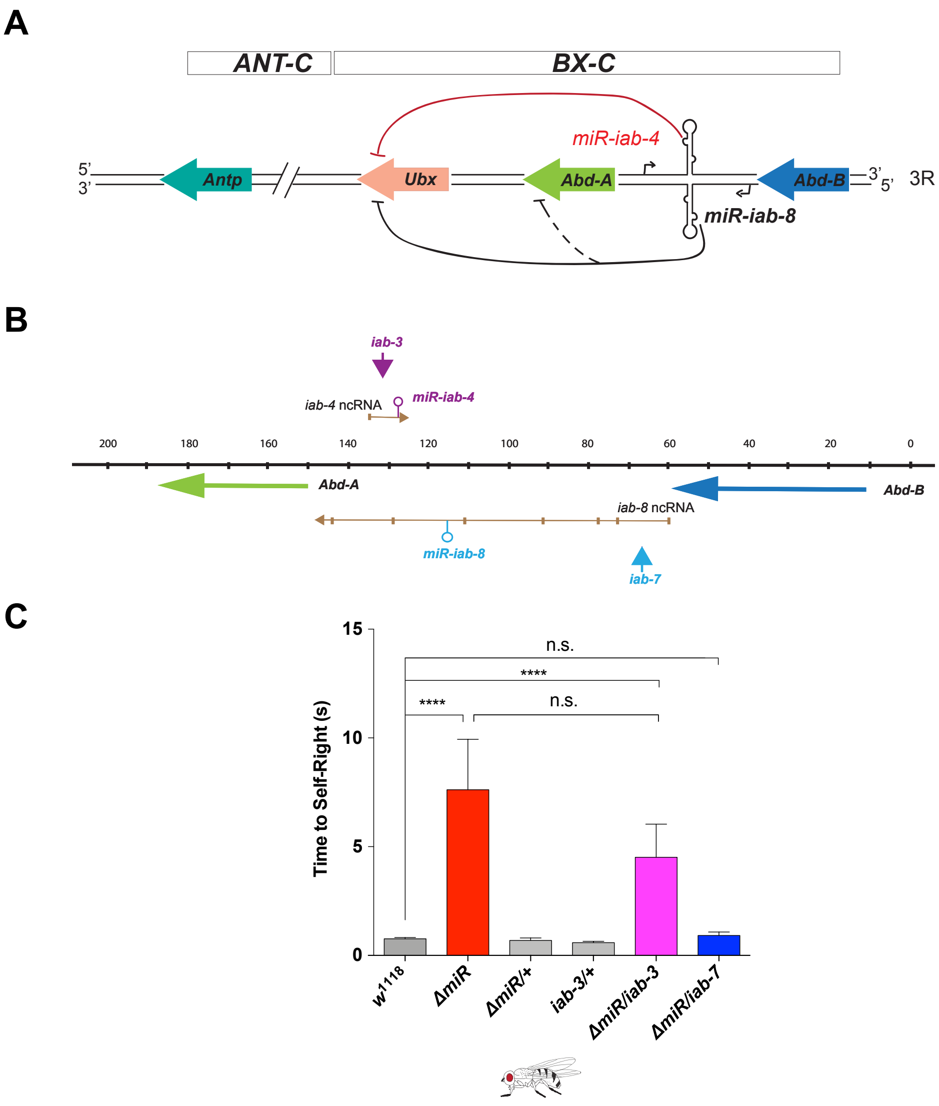

Figure S3. **Genetic complementation tests determine that loss of *miR-iab4* leads to the adult SR phenotype. Related to Figure 1.** (**A**) Diagram of the *Drosophila Hox* complexes (Antennapedia (ANT-C) and Bithorax (BX-C) showing the genomic location of the *Hox* genes *Antp*, *Ubx*, *abd-A* and *Abd-B* and the miRNA system *miR-iab4/8*. Note that transcription of *miR-iab4* and *miR-iab8* occurs from opposite DNA strands. (**B**) Diagram of a sub-region of the *Bithorax* (*BX-C*) complex showing *miR-iab-4* (magenta) and *miR-iab-8* (blue) non-coding RNAs (ncRNA), and breakpoints of rearrangement affecting *miR-iab-4* (iab-3, magenta) and *miR-iab-8* (iab-5 and iab-7, blue). (**C**) Genetic complementation using trans-heterozygote flies for *ΔmiR* and a series of chromosomal rearrangement breakpoints (*iab-3* and *iab-5* or *iab-7*) establish that *miR-iab-4* (and not *miR-iab8*) underlies SR effects in the adult (mean ± SEM; n = 15-50). One-way ANOVA with the post hoc Tukey-Kramer tests were performed to compare treatments; P > 0.05 (nonsignificant; n.s.) and P < 0.001 (***). [N.B: Experiments in adult flies were conducted on wingless specimens (see Materials and Methods and legend of Figure 1)].

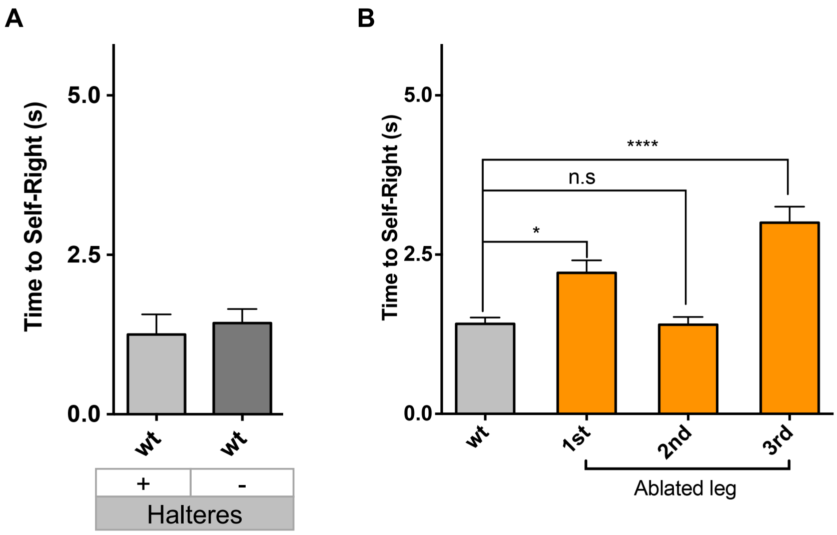

Figure S4. **Ablation experiments. Related to Figure 2.** (**A**) No detectable role of halteres in adult SR. Comparison of SR time in adult wt flies with and without halteres shows no statistically-significant effect of halteres in adult SR (mean ± SEM; n = 12-15). A nonparametric Mann-Whitney U test was performed to compare treatments; $P > 0.05$. [N.B: Adult experiments were conducted on wingless flies (see Materials and Methods)]. (**B**) The role of individual leg pairs in adult SR behaviour. Quantification of the time to complete the SR response in adult wt flies without T1, T2 and T3 leg pairs. Note that T1 and T3 legs lead to significant changes in normal SR time while removal of T2 legs produces no detectable effects on SR (mean ±SEM; N= 40 flies). A nonparametric Mann-Whitney U test was performed to compare treatments; $P > 0.05$ (non significant; n.s.), $P < 0.05$ (*) and $P < 0.001$ (***). [N.B: Experiments in adult flies were conducted on wingless specimens (see Materials and Methods and legend of Figure 1)].

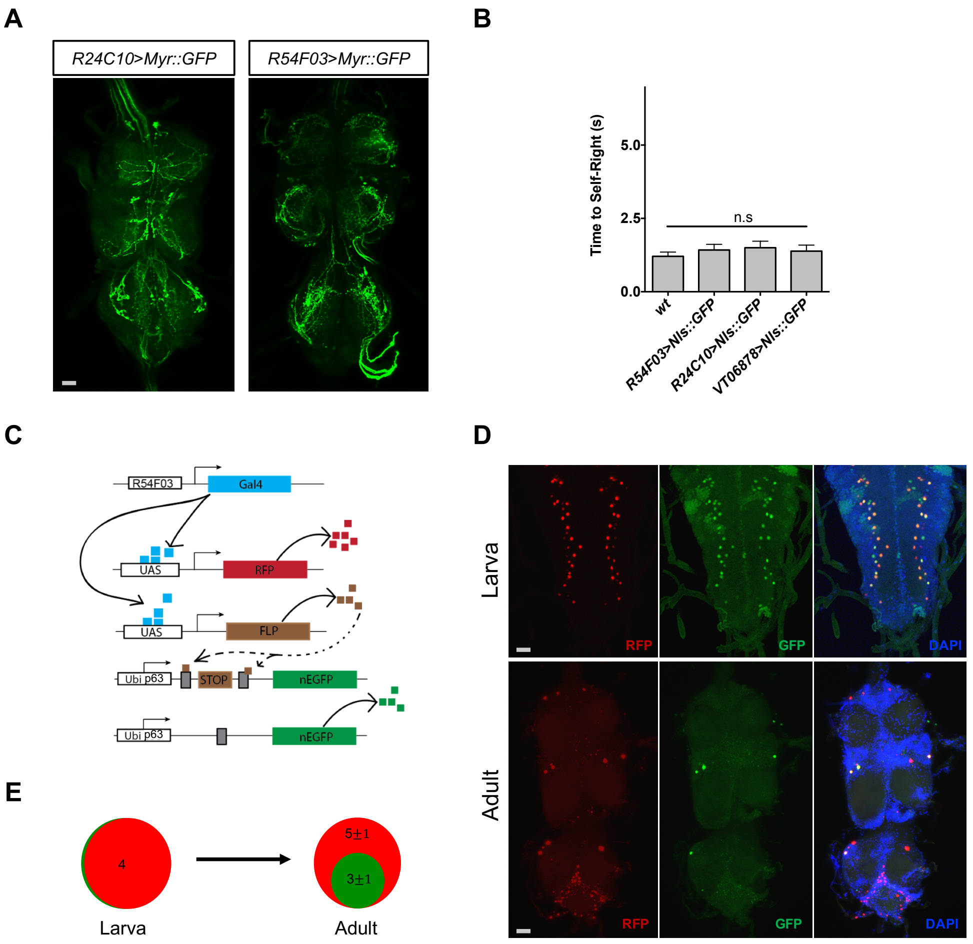

Figure S5. **Roles of specific motor neuron subpopulations in adult SR behaviour. Related to Figure 3.** (**A**) Gal4 driver expression (detected by UAS-GFP) related to tools used in Figure 3. Adult VNC expression of membrane-associated GFP (Myr::GFP) under control of R54F03-Gal4 (*R54F03> Myr::GFP*) or R24C10-Gal4 (*R24C10> Myr::GFP*). (**B**) Flies that express GFP in distinct neuronal populations do not show significant changes in SR times compared with control wt (w^{1118}) flies (mean ± SEM; n = 41). A nonparametric Mann-Whitney U test were performed to compare treatments; P > 0.05 (nonsignificant; n.s.). (**C** and **D**) . Activation of the neural *R54F03* enhancer in the larva and adult VNC. Schematics of the G-TRACE system (**C**). On its first activation, the R54F03 enhancer leads to expression of the Gal4 transgene (Blue) which induces expression of both, a UAS-RFP construct (Red) and a UAS-flipase construct (brown). In turn, flipase activity leads to excision of a stop cassette downstream of a ubiquitin p63-derived promoter leading to expression of nuclear-GFP (green). The system therefore allows comparison of historical (GFP) and actual (RFP) expression driven by the Gal4-driver. G-TRACE data from *R54F03* enhancer activity in larval and adult VNC (**D**). Historical (GFP) and actual (red) expression driven by the *R54F03* driver. (**E**) G-trace analysis across the larval-adult transition shows that R54F03 activity is still on in the adult neuronal populations and that includes all larval derivatives plus an additional population of cells. In the larva, actual/real-time (red)and historical (GFP) neurons represent 4 neurons per segment, and in the adult 5±1 (actual) and 3±1 (historical/lineage) roughly. [N.B: Experiments in adult flies were conducted on wingless specimens (see Materials and Methods and legend of Figure 1)].

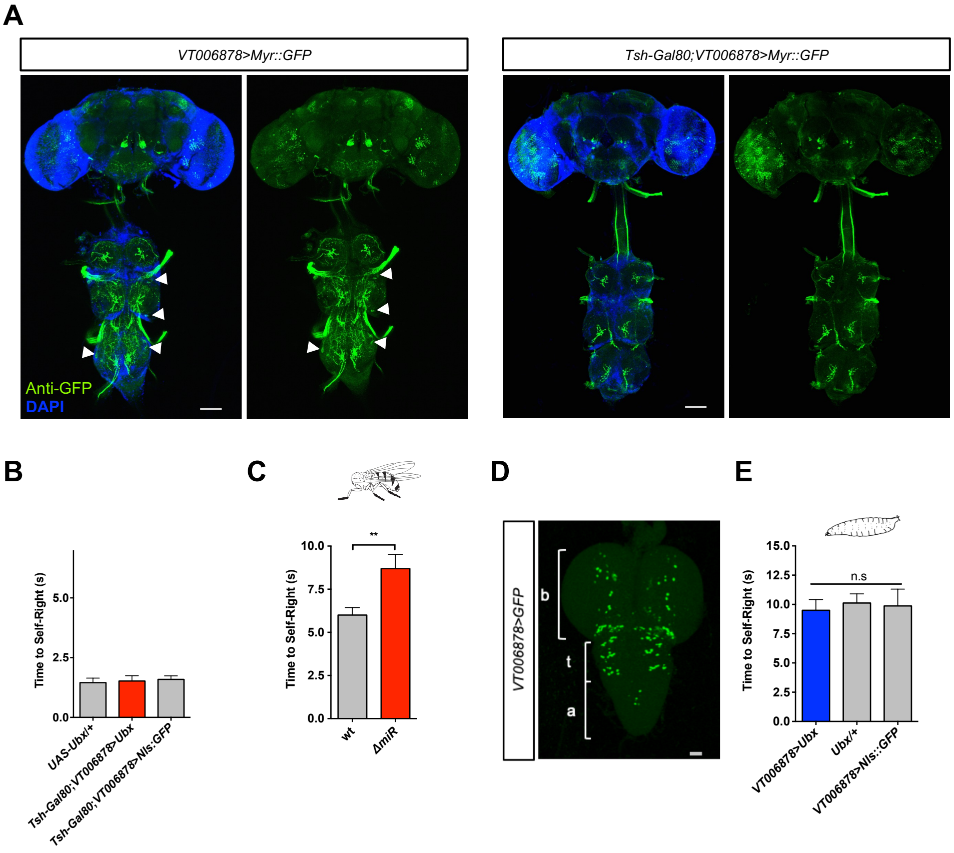

Figure S6. **Upregulation of *Ubx* within the VT006878 domain in the brain is insufficient to generate a SR defects. Related to Figure 3.** (**A**) Maximum intensity projections of *VT006878-Gal4* (green) expression patterns in the presence of *Tsh-Gal80* which represses expression in the entire ventral nerve cord. The pattern of *VT006878-Gal4* in the absence (*VT006878-Gal4> Myr::GFP*) (+/+; *VT006878-Gal4,+ / UAS-Myr::GFP,+*) (top left) and the presence (*Tsh-Gal80*; *VT006878-Gal4> Myr::GFP*) (*Tsh-Gal80*/ +; *VT006878-Gal4,+ / UAS-Myr::GFP,+*) (bottom right) of *Tsh-Gal80* (Arrows highlight neurons somata). The DAPI (blue) is used as neuropil stain. Scale bars for anatomic images, 50 µm. (**B**) Expression of *VT006878>Ubx* (red) in the presence of *Tsh-Gal80* (*Tsh-Gal80*; *VT006878-Gal4>Ubx*) (*Tsh-Gal80*/ +; *VT006878-Gal4,+ / UAS-Ubx,+*) (which represses expression in the entire ventral nerve cord, VNC) leads to no statistically significant changes in SR times when compared with control lines (*UAS-Ubx/+*) (grey) and *Tsh-Gal80*; *VT006878> Nls::GFP* (grey) (mean ± SEM; n = 22). One-way ANOVA with the post hoc Tukey-Kramer (Figure S6E) test were performed to compare treatments (P > 0.05). (**C**) miRNA mutation has statistically significant effects on SR time in decapitated adult flies (mean ±SEM; N= 13 flies). A nonparametric Mann-Whitney U test was performed to compare treatments; P < 0.01 (**). (**D**) VT006878 domain of expression *VT006878>Nls::GFP* in the larval CNS. [Abbreviations: b, t and a are respectively brain, thoracic and abdominal segments]. (**E**) Upregulation of *Ubx* within the VT006878 domain in the larval central nervous system is insufficient to generate a SR defects in larva. (mean ±SEM; N= 16-20 flies). A nonparametric Mann-Whitney U test was performed to compare treatments; P > 0.05.

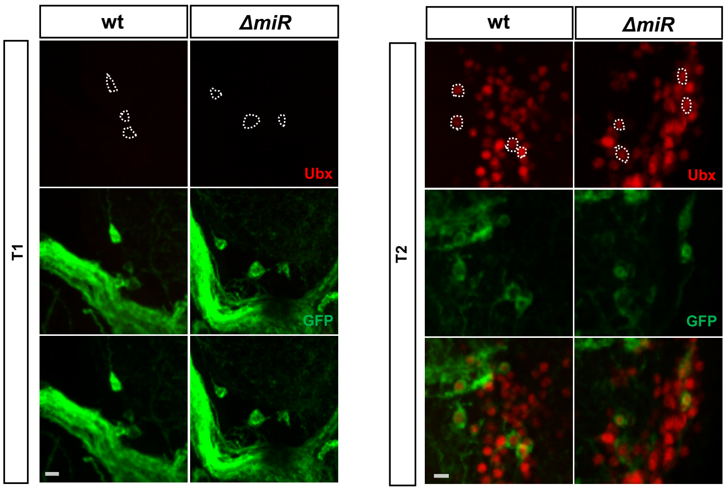

Figure S7. Expression pattern of Ubx protein within the VT006878 domain in the T1 and T2 ganglia of the VNC in wild type and miRNA mutants. Related to Figure 4. There is no expression of Ubx protein within the VT006878 domain in the T1 ganglion (Left). Expression of Ubx protein within the VT006878 domain in the T2 ganglion of normal and mutant adult flies shows no differences in expression across the genotypes (Right).
